# Surgery-Related Quality of Life of Pediatric Patients With Crohn's Disease

**DOI:** 10.3389/fped.2020.608370

**Published:** 2020-12-17

**Authors:** Valeria Dipasquale, Enrica Antonelli, Laura Cannavò, Giorgio Cavatoi, Carmelo Romeo, Giuseppe Trimarchi, Giuseppe Navarra, Claudio Romano

**Affiliations:** ^1^Pediatric Gastroenterology and Cystic Fibrosis Unit, Department of Human Pathology in Adulthood and Childhood “G. Barresi”, University of Messina, Messina, Italy; ^2^Pediatric Surgery Unit, Department of Human Pathology in Adulthood and Childhood “G. Barresi”, University of Messina, Messina, Italy; ^3^Department of Economy, University of Messina, Messina, Italy; ^4^Surgical Oncology Division, Department of Human Pathology in Adulthood and Childhood “G. Barresi”, University of Messina, Messina, Italy

**Keywords:** Crohn's disease, inflammatory bowel disease, health-related quality of life, surgery, questionnaire, pediatrics

## Abstract

**Objective:** Up to 30% of pediatric patients with Crohn's disease (CD) require surgery. The aim of the study was to evaluate long-term health-related quality of life (HRQoL) outcome in children with CD who have had ileocolonic resection.

**Materials and methods:** This was a retrospective cross-sectional study on all pediatric patients who had undergone surgery for CD between January 2015 and December 2017 in the Pediatric Surgery and Gastroenterology Units of the University Hospital of Messina. Surgical treatment was represented by laparoscopic ileocecal resection with latero-lateral anastomosis. Patients were asked to fill in a modified version of the IMPACT III questionnaire made up of 15 closed questions before and after surgery. The questionnaire was scored on a five-point scale with 5 reporting “not a problem” and 1 “a very severe problem.” The total score ranged from 15 (worst HRQoL) to 75 (best HRQoL). Frequency of relapses, reoperations, complications during follow-up, and postoperative bowel function were also studied.

**Results:** Data were obtained in 10 patients (9 males), who underwent surgery at a median age of 13.5 years (range 13–18), after a median post-diagnosis period of 2.5 years (range 0–8). Preoperative scores were low in all 4 domains of the questionnaire. Postoperatively, HRQoL measures improved significantly (*p* < 0.05) about symptoms, school attendance, social and emotional functioning. Overall, nearly all patients were completely satisfied with the surgical outcome.

**Conclusions:** HRQoL is low in CD children referred for possible operation, and surgery may positively affect the overall HRQoL. Collecting HRQoL data provides insight into the impact of treatment on children health status.

## Introduction

Crohn's disease (CD) is a chronic, relapsing disease whose symptoms interfere with the daily activities of patients, especially in young patients who have a long life expectancy (1, 2). The incidence of pediatric CD, particularly in children 10–19 years of age, is increasing and the phenotype is often characterized by an aggressive disease course, higher risk of surgery (because of stenosis), and more extensive phenotypes compared with adults (1, 3). Quality of life (QoL) is considered the most important outcome for chronic conditions such as CD. In general, QoL is the feeling of overall life satisfaction, as determined by the mentally alert individual whose life is being evaluated. In health care, health-related QoL (HRQoL) is the assessment of how the individual's well-being may be affected by a disease, disability, or disorder (4, 5). Numerous physical and psychological factors contribute to the poor HRQoL in pediatric patients with CD, such as its relatively young age of onset, associated extraintestinal complications, disability, and debilitating symptoms (diarrhea, abdominal pain, gastrointestinal bleeding) that have the potential to cause significant psychosocial stress (1, 6). Even in the era of routine immunomodulator and biological therapies for moderate to severe CD, modifying the natural history of the disease remains a challenge, and about 20–30% of children require surgery within 10 years of diagnosis (1, 7, 8). Indications for surgery in CD include complications such as stricture, perforation, abscess, fistula, or severe perianal disease (1). Patients being referred for surgery experience a more aggressive disease and a poorer HRQoL (1, 9). Literature data mainly coming from adult studies showed that HRQoL in these patients seems to improve following resectional surgery (6, 10, 11). In pediatric patients with CD, the effects of small bowel resection on HRQoL have not been systematically studied. The aim of the present study was to evaluate the long-term health outcomes of children with CD and undergone ileocolonic resection and to compare preoperative and postoperative HRQoL with a disease-specific questionnaire.

## Materials and Methods

### Patients

This retrospective cross-sectional study included all the pediatric patients (18 years or younger) followed up and undergone surgery for CD in the Pediatric Surgery, Pediatric Gastroenterology, and Adult Surgery Units of the University of Messina. Hospital records from January 2015 to December 2017 were reviewed. Patients with <1 year of follow-up were excluded. Data on demographics, age at diagnosis of CD, age at the time of surgery, the medication before surgery, indications for surgery, postoperative use of biologics, and possible surgical complications and re-resections were recorded from case notes. The diagnosis of CD was based on clinical features, laboratory tests, and endoscopic and histological findings. Disease activity was reported based on the Pediatric Crohn's Disease Activity Index (PCDAI) (12). Surgery was represented by laparoscopic-assisted ileocolonic resections with latero-lateral anastomosis. Disease relapse after surgery was defined as the presence of aphthous lesions according the Rutgeerts score (13) to predict postsurgical CD recurrence via ileocolonoscopy. This study was conducted in conformity with the principles and regulations of the Helsinki Declaration. The study was approved by the committee on research ethics of all participating sites and informed consent from participants and their parents, where necessary, was obtained.

### Questionnaire

The instrument chosen to measure HRQoL in children affected by CD before and after surgery was a modified version of the IMPACT III questionnaire (14), which is a specific inflammatory bowel disease (IBD) questionnaire assessing the quality of life in an objective dimension. We modified the IMPACT III by decreasing the number of issues in order to make it easier to be completed by phone interview. The final version was made up of 15 closed questions of functional outcomes and HRQoL. All of the issues were grouped into 4 domains, such as systemic and IBD symptoms (7 questions), school functioning (2 questions), social functioning (3 questions), emotional functioning (3 questions) (Supplementary Table 1). The questionnaire was scored on a five-point scale with 5 reporting “not a problem” and 1 “a very severe problem.” The total score ranged from 15 (worst HRQoL) to 75 (best HRQoL). The questionnaire also included an initial question asking the participants to comment on general satisfaction with surgery. Questionnaires were administered by the physician (pediatric surgeon or pediatric gastroenterologist) by telephone interview at 12 and 6 months before surgery and at 12 months following surgery. Respondents were patients or parents in case of minor patients.

### Outcomes

Primary outcome was postoperative long-term HRQoL. Frequencies of relapses, reoperations, complications during follow-up, and postoperative bowel function were also studied.

### Statistical Analysis

Continuous variables were presented as means and SD while the categorical variables were reported as percentages. The Shapiro–Whilk test showed variable distribution was asymmetric. The non-parametric Friedman test for paired data and the Conover *post-hoc* analysis (15) were used to compare the study times (6 months before vs. 12 months after surgery). Statistical analyses were performed in R version 3.5.3 (16) using the PMCMR plus (Calculate Pairwise Multiple Comparisons of Mean Rank Sums Extended) package. A *p* < 0.05 was considered significant.

## Results

Data were obtained in 10 patients (9 males), who underwent surgery at a median age of 13.5 years (range 13–18). The median amount of time that had elapsed after the operation was 4 years (range 1–9). The majority of patients underwent surgery because of active disease despite optimal medical therapy (4 with anti-TNF alpha). Table 1 presents the background data of the patients.

**Table 1:** Patients' demographic and clinical variables.

**Variable**	**Total (*N* = 10)**
Current age, y, median (range)	17.2 (12–23)
Age at diagnosis, y, median (range)	12.1 (6–16)
Ileocolonic disease, *n*	10
Disease duration before operation, y, median (range)	2.5 (0–8)
Age at surgery, y, median (range)	13.5 (13–18)
Follow up time after operation, y, median (range)	4 (1–9)
Moderate disease activity 12 months before surgery, n^*^	9
PCDAI score 12 months before surgery, median	22
Moderate disease activity 6 months before surgery, n^*^	10
PCDAI score 6 months before surgery, median	25
Remission 12 months after surgery, n^**^	10
PCDAI score 12 months after surgery	9
**Indications for surgery**, ***N***	
Bowel stricture	7
Bowel stricture with enteroenteric fistula	3
**Medications at surgery**, ***N***	
Azathioprine	4
Anti-TNF (IFX, ADA)	4
None	2

* *Pediatric Crohn's Disease Activity Index score 11–30*.

** *Pediatric Crohn's Disease Activity Index score <10*.

*IFX, infliximab; ADA, adalimumab*.

HRQoL median scores were 43.2 and 34.5, 12 and 6 months before surgery, respectively. There was a significant improvement seen at 12 months postoperatively (median 65.4, *p* < 0.001). This improvement was observed in all dimensions of HRQoL. About systemic and gastrointestinal symptoms (Table 2), a highly significant reduction (*p* < 0.001) of stool blood, abdominal pain, and fatigue was reported at 12 months after surgery. A significant improvement (*p* < 0.001) of appetite and weight was also reported, with less difficulty to perform the general activity of daily living (*p* < 0.001). Nonetheless, despite a slight reduction, stool frequency improvement was not significant in comparison to baseline.

**Table 2:** Systemic and IBD symptoms score preoperatively and 6 and 12 months after surgery.

**Issue**	**12 months before surgery (*n* = 10)**	**6 months before surgery (*n* = 10)**	**12 months after surgery (*n* = 10)**	p^*^
Stool blood, median	3.40 ± 1.26	3.20 ± 1.32	4.60 ± 0.70	<0.001
Increased stool frequency, median	4.80 ± 0.63	4.60 ± 0.84	5	0.223
Abdominal pain, median	2.20 ± 1.03	1.40 ± 0.84	4.60 ± 0.84	<0.001
Fatigue, median	1.40 ± 0.84	1	4.40 ± 0.97	<0.001
Reduced caloric intake, median	3.00 ± 1.89	2.20 ± 1.40	5	<0.001
Weight loss, median	2 ± 1.41	1.40 ± 0.84	4.80 ± 0.63	<0.001
Need to stop daily activities, median	3 ± 1.63	1.80 ± 1.40	4.60 ± 0.84	<0.001

* *Comparison between 6 months before and 12 months after surgery*.

*IBD, Inflammatory bowel disease*.

Nearly all patients reported frequent school absences before surgery, with low scores both at 12 (median score 2.60 ± 1.26) and 6 months (1.60 ± 0.97). The most reported IBD-related problems which had caused an absence from school were abdominal pain, fatigue and diarrhea in 5, 3, and 2 patients both 12 and 6 months before surgery. School absences significantly reduced at 12 months after surgery (4.60 ± 0.84, *p* < 0.001).

Preoperatively, the scores showed markedly impaired social functioning (Table 3), including the ability to play and/or go out with friends, perform a sport, travel and/or go on holiday, with a significant recovery at 12 months from surgery (*p* < 0.001, *p* < 0.008, *p* < 0.002, respectively).

**Table 3:** Social activities score preoperatively and 6 and 12 months after surgery.

**Issue**	**12 months before surgery (*n* = 10)**	**6 months before surgery (*n* = 10)**	**12 months after surgery (*n* = 10)**	p^*^
Play and/or go out with friends, median	2.20 ± 1.40	1.60 ± 0.97	4.40 ± 1.35	<0.001
Play a sport, median	2.40 ± 1.35	1.80 ± 1.40	4 ± 1.41	<0.008
Travel and/or go on holiday, median	3.60 ± 1.65	2.60 ± 1.58	4.80 ± 0.63	<0.002

* *Comparison between 6 months before and 12 months after surgery*.

About the emotional functioning (Table 4), CD patients reported a significant reduction of feelings of anger, injustice and embarrassment due to their disease at 12-month follow-up (*p* < 0.036 and *p* < 0.039, respectively). On the contrary, surgery did not affect their concerns for future health problems (*p* = 0.202). Overall, nearly all patients were completely satisfied (score 5) with the outcome of the surgery at 12-month follow-up, with absolute score values of 75.

**Table 4:** Emotional functioning score preoperatively and 6 and 12 months after surgery.

**Issue**	**12 months before surgery (*n* = 10)**	**6 months before surgery (*n* = 10)**	**12 months after surgery (*n* = 10)**	p^*^
Feelings of anger or injustice related to the disease, median	3 ± 1.89	2.40 ± 1.65	4 ± 1.05	<0.036
Concerns for the future, median	4 ± 1.05	3.60 ± 1.35	4.20 ± 1.03	0.202
Embarrassment because of bowel condition, median	3.40 ± 1.84	3.20 ± 1.75	4.40 ± 1.35	<0.039

* *Comparison between 6 months before and 12 months after surgery*.

There were no operation-related mortality and complications. Two patients were started on biologics (adalimumab) for preventing postoperative recurrence. By the end of the follow-up, all patients were in clinical remission (PCDAI <10), and none of them experienced surgical complications and/or recurrences (Rutgeerts score <i2).

## Discussion

Surgery has a definite role in CD treatment protocols, and seems to positively impact the natural history of the disease (17). Some studies have demonstrated that the early use of biologics do not reduce the risk of surgery in CD (1, 18). Surgery can be a viable option in elective cases of CD, such as stricture or fistula, growth retardation, delayed puberty or a clinical condition unresponsive to medical treatment. It is also to be considered in urgent situations, such as occlusive syndrome, abscess or fistulas unresponsive to conservative treatment, massive hemorrhage or intestinal perforation (17).

In the present retrospective cross-sectional study, the long-term HRQoL and health-outcomes after surgery in pediatric CD median 4 years after bowel resection were studied, and it was showed that children and adolescents with CD have low HRQoL score and that HRQoL significantly improves postoperatively. A very limited number of studies evaluated the HRQoL in pediatric patients before and after surgery for CD (9, 19). In 2013 Piekkala et al. (19) carried out a retrospective study on 36 pediatric CD patients who had undergone intestinal resections during childhood between 1985 and 2008. Moreover, they selected 2 controls (with no IBD) per patient with CD matched for age and sex. All patients and controls answered questionnaires about health outcomes and QoL. Most patients had undergone surgery because of active disease despite optimal medical therapy (3 with infliximab) or steroid dependency (56%), as in the present study. The median follow-up after primary resection was longer than our study, with a median of 10 years (range 2–21). The different dimensions and overall HRQoL scores were similar between the patients and their matched controls. Similarly to our study, 96% were completely or moderately satisfied with the outcomes of the surgery (19). Notably, the overall HRQoL, physical and social functioning at follow-up were significantly lower among patients who had missed school or work because of surgery compared with patients who could normally attend school or work. Two other studies performed in the same center on limited number of patients (5 in each study) reported restricted sport activities in children due to stoma formation (20, 21). Permanent stoma had no impact on HRQoL in children compared to adults with a stoma who scored lower for general health and physical activity compared to controls (19, 22). In this study, no surgical complications were registered during the follow-up period. Data coming from pediatric literature are scarce and suggest that the number of complication in pediatric patients are higher if compared with adult data, but further studies are warranted. In the study by Piekkala et al. (19), nearly all patients had at least 1 surgical complication, mostly represented by strictures in the anastomosis.

About disease reactivation after surgery, literature data consistently report that about 50–60% of pediatric patients had a clinical relapse within 1.8–5 years postoperatively (7, 19, 23–26). On the other hand, the reoperation rates are very variable, ranging from 8–18% at 2 years (7, 27) to 29–50% at 10 years (17, 27). In this study, no disease re-activation after surgery was recorded, probably due to the small number of patients. One patient was started on anti-TNFα therapy soon after the intervention, as recommended in high-risk patients (extensive disease, short disease duration from diagnosis to surgery, recurrent surgery, long resected segment, surgery for fistulizing disease, disease complications, perianal disease, smoking) (1, 28).

Any conclusions drawn from the findings of this research study must be qualified in light of the study's limitations. First, the small number of patients, which precluded reliable statistical analysis (such as the evaluation of possible predictors of HRQoL after surgery); second, the retrospective design, so recruited cases and clinical management are the confounding variables; third, the lack of a control population, in order to compare life situation (and HRQoL) with that of patients with CD. Moreover, data were obtained by phone and not during a face to face consultation. The strength of the present study is being one of the limited number of studies reporting HRQoL outcome before and after surgery for CD in pediatric patients. Collection of HRQoL data provides insight into the impact of treatment on patient health status and well-being, especially in the pediatric group of age.

In conclusion, this study confirms that HRQoL is low in children and adolescents with complicated CD who previously failed medical therapy. Ileocecal resection for CD is safe and associated with significant and sustained improvement of the physical, psychological, and social dimensions of HRQoL in affected children. CD is a model of chronic disease, whose clinical and pharmacological management is crucial to gain an optimal HRQoL. On the other hand, the impact of therapy on HRQoL should be considered an integrative, “positive” step of the management strategy. The clinician should consider these HRQoL-related aspects, in comparison with pharmacological therapies-related concerns, such as the high infection risk carried out by high dose of biological or immunosuppressant drugs. This present study adds a contribution in this area, and confirms that surgery may positively affect HRQoL in pediatric patients with CD.

## Data Availability Statement

The raw data supporting the conclusions of this article will be made available by the authors, without undue reservation.

## Ethics Statement

The studies involving human participants were reviewed and approved by Ethics Committee of the University of Messina. Written informed consent to participate in this study was provided by the participants' legal guardian/next of kin.

## Author Contributions

All authors have made substantial contributions to the design of the study, acquisition and interpretation of data, drafting the article and revising it critically for important intellectual content, and finally approved the version to be submitted.

## Conflict of Interest

The authors declare that the research was conducted in the absence of any commercial or financial relationships that could be construed as a potential conflict of interest.
