# Supplementary material for: Surgery-Related Quality of Life of Pediatric Patients With Crohn's Disease
Source: Front Pediatr. 2020 Dec 17;8:608370. doi: 10.3389/fped.2020.608370 (PMC7773926; doi:10.3389/fped.2020.608370)
Supplement: Supplementary file 1 [file Table_1.DOCX]

Supplementary Material

**Supplementary Table 1.** Questionnaire questions

| Questions* |
| --- |
| 1) Are you satisfied with the final results of the surgical procedure? |
| *Is each of the following a problem, for you? If yes, how much?* |
| 2) Stool blood |
| 3) Increased stool frequency |
| 4) Abdominal pain |
| 5) Fatigue |
| 6) Reduced caloric intake |
| 7) Weight loss |
| 8) Need to stop daily activities |
| 9) School absence |
| *Do you have any difficult in doing each of the following? If yes, how much?* |
| 10) Play and/or go out with friends |
| 11) Play a sport |
| 12) Travel and/or go on holiday |
| *Do you experience one of the following? If yes, how often?* |
| 13) Feelings of anger or injustice related to the disease |
| 14) Concerns for the future |
| 15) Embarrassment because of bowel condition |

* The questionnaire was scored on a five-point scale with 5 reporting not a problem and 1 a very severe problem
